# Repertoire of Protein Kinases Encoded in the Genome of *Takifugu rubripes*

**DOI:** 10.1155/2012/258284

**Published:** 2012-05-14

**Authors:** R. Rakshambikai, S. Yamunadevi, K. Anamika, N. Tyagi, N. Srinivasan

**Affiliations:** ^1^Molecular Biophysics Unit, Indian Institute of Science, Bangalore 560012, India; ^2^Max Planck Institute for Intelligent Systems (Formerly Max Planck Institute for Metals Research), BioQuant BQ0038, Im Neuenheimer Feld 267, 69120 Heidelberg, Germany; ^3^German Cancer Research Center, BioQuant BQ0038, Im Neuenheimer Feld 267, 69120 Heidelberg, Germany; ^4^Department of Functional Genomics and Cancer and Department of Structural Biology and Genomics, Institut de Génétique et de Biologie Moléculaire et Cellulaire (IGBMC), CNRS UMR 7104, INSERM U 964, Université de Strasbourg, 1 rue Laurent Fries, 67404 Illkirch Cedex, France; ^5^European Bioinformatics Institute, Wellcome Trust Genome Campus, Hinxton, CB10 1SD Cambridge, UK

## Abstract

*Takifugu rubripes* is teleost fish widely used in comparative genomics to understand the human system better due to its similarities both in number of genes and structure of genes. In this work we survey the fugu genome, and, using sensitive computational approaches, we identify the repertoire of putative protein kinases and classify them into groups and subfamilies. The fugu genome encodes 519 protein kinase-like sequences and this number of putative protein kinases is comparable closely to that of human. However, in spite of its similarities to human kinases at the group level, there are differences at the subfamily level as noted in the case of KIS and DYRK subfamilies which contribute to differences which are specific to the adaptation of the organism. Also, certain unique domain combination of galectin domain and YkA domain suggests alternate mechanisms for immune response and binding to lipoproteins. Lastly, an overall similarity with the MAPK pathway of humans suggests its importance to understand signaling mechanisms in humans. Overall the fugu serves as a good model organism to understand roles of human kinases as far as kinases such as LRRK and IRAK and their associated pathways are concerned.

## 1. Introduction

*Takifugu rubripes* is a teleost fish native to northwest pacific seas. It belongs to the family Tetraodontidae and order Tetraodontiformes. The fugu genome is rather compact with a size of ~400 Mb although the number of genes is comparable to that of higher eukaryotes indicating a considerable reduction in the intergenic regions [1]. Detailed analysis reveals that the intron-exon boundaries [2] and in certain cases alternative splicing [3], synteny [4, 5] have been conserved with respect to that of humans suggesting the possibility of conserved elements from a common ancestor. Thus, due to such features indicating close relationship, the fugu is suggested to be a good model organism and an effective way to study evolution of structure of complex vertebrate genomes [4, 6].

 In 2002, the first draft sequence of the fugu genome was reported by the International fugu genome Consortium using the “whole-genome shotgun” strategy. Subsequently many versions of the genomic data have been made available at http://www.fugu-sg.org/. The latest version (v-5) was released in 2010 which covers about 392 Mb and 72% of genome being organized into chromosomes.

Response to environmental stimulus via complex signaling systems is a central feature of all living cells. Phosphorylation is one such posttranslational modification employed in signaling circuits which usually results in a functional change in the substrate by changing enzyme activity, cellular location, or association with other proteins. Thus, protein kinases have implication in regulation of various cellular processes encompassing metabolism, stress responses, cell cycle control, organ development, and intercellular communication [28, 29]. Abnormalities in the functioning of these kinases usually have implications in developmental disorders and malignancies [30, 31]. The eukaryotic kinases mainly constitute the Ser/Thr and Tyr kinases which share a common three-dimensional fold and the catalytic core spanning to about 300 residues [32].

The fugu genome has been used in a myriad of comparative genomic studies to elucidate the function of proteins involved in neurodegenerative diseases [33, 34], signaling systems [35, 36], and so forth and has been suggested as a method to elucidate cognate pathways in humans. In this paper, using sensitive sequence analysis [37–54] we recognize the repertoire of Ser/Thr and Tyr kinases encoded in the fugu genome. This is not trivial as homologous kinases are known to be characterized by weak sequence similarity. In addition, we classify these kinases on the basis of their catalytic domain sequence and the domains covalently tethered to the catalytic kinase domain [38]. Finally, we provide comparative analysis with distribution of the kinases in other model organisms and other proteins of the MAPK pathway especially in relation to the higher eukaryotes like human.

## 2. Materials and Methods

### 2.1. Identification of Protein Kinases

**Figure 1:**
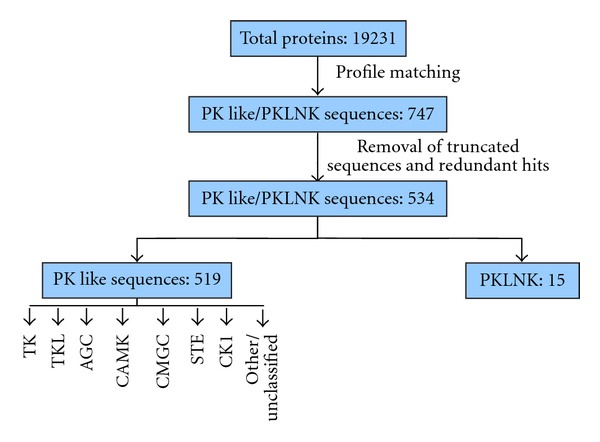
Distribution of fugu kinases into various Hanks and Hunter groups. PKLNK: protein-kinase-like nonkinases; CAMK: calcium/calmodulin-dependent protein kinase; CMGC: the group of cyclin-dependent protein kinase, mitogen-activated protein kinase, glycogen synthase kinase, casein kinase-2; AGC: the group of protein kinase A, protein kinase G, protein kinase C; STE: sterile (homologs of yeast STE); TK: tyrosine kinase; TKL: tyrosine kinase like; CK1: casein kinase 1.

The complete set of predicted proteins from the ORF's of the *Takifugu rubripes* fifth assembly genome has been obtained from http://www.fugu-sg.org/. We have adopted sensitive sequence profile matching algorithms to identify and examine Ser/Thr and Tyr kinases encoded in the genome. The protocol used is identical to that adopted for analysis of kinases of other organisms earlier in this laboratory [43–49]. Briefly, we have employed multiple sensitive sequence search and analysis methods PSI-BLAST [37], MulPSSM [39–41] involving extensive use of RPS-BLAST [41] and HMMer [42] which match Hidden Markov Models (HMMs) to identify protein kinase catalytic domain and their co-occurring domains. The criteria used to associate a given protein kinase to a given subfamily on the basis of its primary structure include the degree of sequence identity greater than 30% with members of known subfamily of kinases and the presence of signature amino acids that are characteristics of protein kinase subfamilies [43] which include the glycine rich loop and catalytic aspartate of consensus sequence HRDLKXXN. In addition, search procedures such as PSI-BLAST have been used to detect sequences homologous to the kinase catalytic domain using an *E*-value cutoff of 0.0001 which is decided on the basis of previous prototypic study [50]. Truncated sequences which are less than 200 amino acids long were eliminated to arrive at a set of 534 PK- (protein-kinase-) like sequences. The data set of putative PK-like sequences has been obtained from the compilation of hits obtained during various search procedures. Out of these, 15 sequences lack aspartate in the catalytic loop and, therefore, are unlikely to function as kinases. These are referred as protein-kinase-like non-kinases (PKLNKs) [55]. These sequences were subjected to fold recognition approach PHYRE (http://www.sbg.bio.ic.ac.uk/phyre/) [51, 52] to ensure that they fold like kinases. The final number of 519 sequences is likely to function as protein kinases. The entire operation works stepwise with filtering of sequences at every stage in order to recognize kinases. The number of sequences involved at various stages is depicted in Figure 1.

### 2.2. Classification of Kinases into Hanks and Hunter Groups

Hanks and Hunter have proposed classification of kinases based on sequence analysis [38]. In order to classify the fugu kinases into these groups and subfamilies reverse PSI-BLAST (RPS-BLAST) was used to search each of the 519 PK-like sequences as a query against the database containing 2810 position-specific scoring matrices (PSSMs) created for the various subgroups of protein kinases corresponding to sub-families of kinases. A query kinase sequence was associated to its subfamily based on the extent of sequence similarity. Sequences with greater than 30% identity and 70% profile coverage with at least one of the members of a kinase groups have been considered as members of the group or subfamily concerned. CLUSTALW [53] was used to generate multiple sequence alignment for the 519 kinases that were associated to specific groups. MEGA version 4 [54] was used to generate the dendrogram showing various groups of protein kinases. The sequences belonging to the group “Others” have been clustered using another dendrogram. Also MEGA 4 [54] has been used to cluster sequences of the kinase domain regions of certain families from other organisms including human, *Drosophila melanogaster, Caenorhabditis elegans, *and* Saccharomyces cerevisiae.*

### 2.3. Assignment of Domains to Multidomain Kinases

Domain assignments have been made for protein kinase catalytic domain containing gene products using the HMMer method by querying each of the kinase domain containing proteins against the protein family HMMs available in the Pfam database [56] and MULPSSM profiles [40, 41] of families in Pfam database. Transmembrane segments were detected using TMHMM [57].

### 2.4. Identification of MAPK Pathway Proteins

Sequences of MAPK proteins involved in the MAPK pathways in human were obtained from KEGG pathway http://www.genome.jp/kegg/pathway.html [58]. The sequences were then used as a query to search using BLAST against the predicted protein sequences of fugu genome with an E-value cut off of 0.0001. The hits were obtained after pruning on the basis of coverage and percentage identity. The proteins which did not identify any homolog were then queried with an integrated dataset of fugu genome and SWISSPROT using PSI-BLAST [37] with an *E*-value cutoff of 0.001.The pathway was then generated as a network using CYTOSCAPE2.6.3 [59].

## 3. Results

The genome of fugu encodes 534 PK-like sequences. Of the 534 PK-like sequences 519 them possess the critical aspartate residue at the location characteristic of catalytic base and have at least one glycine conserved in the “glycine rich” loop GXGXXG present in the subdomain I of the kinase catalytic domain. A list of all these 519 sequences identified in this work along with classification and domain combinations are deposited in the KinG database [43] which was developed in this laboratory and the information is publicly available at http://king.mbu.iisc.ernet.in/. In addition, these 519 kinases are listed in the supplementary data file 1 in Supplementary Material available online at doi. 10.1155/2012/258284. The 15 sequences lacking the critical aspartate are unlikely to be functional as kinases though they are likely to adopt the kinase fold. Though the exact roles of these PKLNK's (protein kinase like non kinases) is not entirely clear, such sequences have been reported to have implications in various signaling pathways [60]. Out of the 519 putative functional kinases, 407 of them have an arginine residue preceding the critical aspartate and these are called as the “RD” kinases [48, 61]. This arginine has been indicated to be to be involved in an interaction with phosphate group of a phosphorylated sidechain in the activation loop of kinases. This interaction is known to be a critical step in switching a kinase from inactive to active state through conformational changes. Hence the switching mechanism of these RD kinases is likely to be mediated by phosphorylation at the activation segment in the kinase catalytic domain.

Among the putative PKs, 135 are likely to be tyrosine kinases and 296 are likely to be Ser/Thr kinases. 17 of the 296 Ser/Thr kinases are predicted to have membrane spanning regions. Similarly, 50 of the tyrosine kinases are predicted to be receptor tyrosine kinases. List of these kinases including the information on predicted transmembrane region is included in the supplementary data file 1.

### 3.1. Groupwise Distribution of Protein Kinases

**Figure 2:**
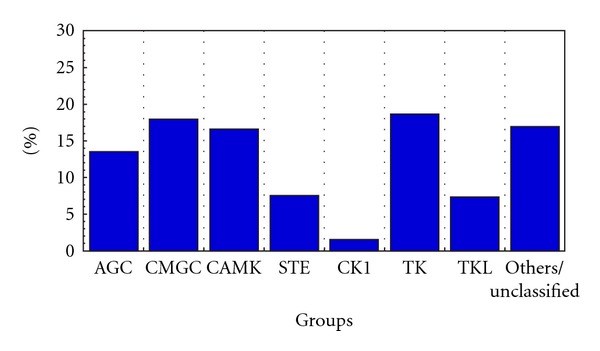
Percentage-wise distribution of fugu kinase groups. CAMK: calcium/calmodulin dependent protein kinase; CMGC: a group of cyclin dependent protein kinase, mitogen activated protein kinase, glycogen synthase kinase and casein kinase-2; AGC: a group of protein kinase A, protein kinase G, and protein kinase C; STE: sterile (homologs of yeast STE); TK: tyrosine kinase; TKL: tyrosine kinase like; CK1: casein kinase 1.

**Figure 3:**
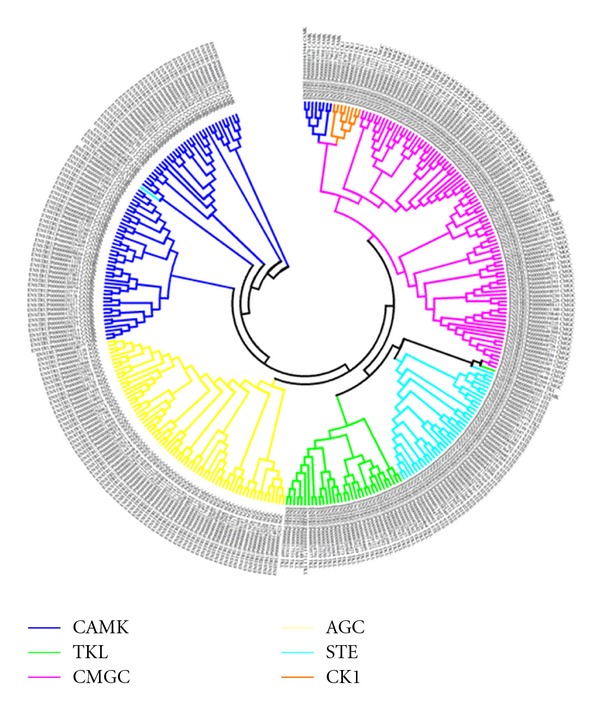
Dendrogram depicting clustering of fugu kinases. Abbreviations followed in the diagram are same as listed in the legend of Figure 2.

**Figure 4:**
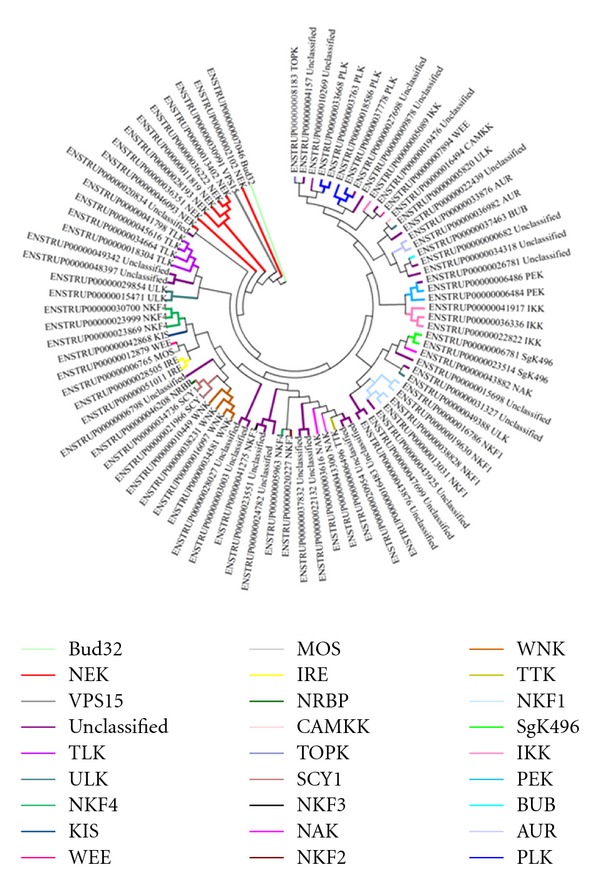
Dendrogram depicting clustering of kinases belonging to the “Other/Unclassified” group.

**Figure 5:**
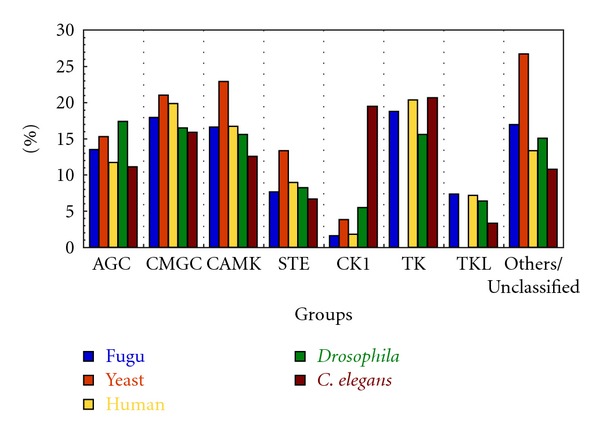
Comparison of group wise distribution of kinases from fugu, human, yeast, *Drosophila* and *C. elegans*. Abbreviations followed in the figure are same as given in the legend to Figure 2.

The 519 kinases have been classified on the basis of the Hanks and Hunter [38] scheme of classifying protein kinases into 7 groups with clearly defined functional roles, namely, AGC (regulated by binding of second messengers), STE (proteins featuring in the MAPK signaling cascades in yeast), CMGC (include MAPK, CDK proteins), CAMK (Calcium/Calmodulin regulated kinases), CK1 (Casein kinase 1), TK (tyrosine kinase), and TKL (tyrosine kinase like). Apart from the 7 groups enlisted, there is yet another group which comprises of sequences that cannot be classified into any of the standard groups and are termed as “Other/Unclassified.” The group-wise distribution of kinases in fugu has been shown in Figure 2 with the TK group being most prevalent and CK1 the least prevalent. Clustering of the sequences of the catalytic kinase domain using BLAST-CLUST was performed and 27 sequences are identified as outliers to the group/subfamily concerned. A dendrogram was constructed without these 27 sequences or the TK group and it resulted in clear distinct grouping of kinases (Figure 3). Though the members belonging to the “Other/Unclassified” group are significantly different from the classical groups, clusters are observed indicating high similarity among the sequences within a node (Figure 4). This points to the possible emergence of newer subfamilies of protein kinases that do not conform to the known groups [38] in the classification of kinases. However it is also possible that some of these “new subfamilies” represent outliers of currently known subfamilies. The group-wise distribution (percentage number of sequences in each group) has been compared with that of other model eukaryotic organisms such as human, *Drosophila melanogaster, Caenorhabditis elegans, *and* Saccharomyces cerevisiae *by performing similar analysis (Figure 5 and Table 1). The distribution of kinases in fugu is very similar to that of other eukaryotes considered and in most cases very similar to that of humans at the group level.

**Figure 6:**
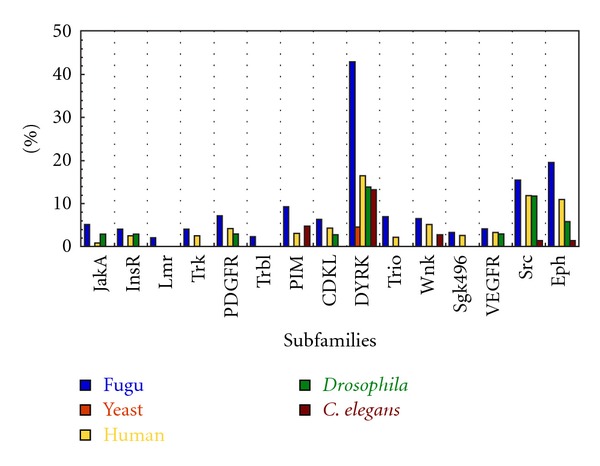
Percentage distribution of subfamilies that are overrepresented in fugu in comparison to various model organisms. JakA: janus kinase A; InsR: insulin receptor, Lmr: lemur kinase; Trk: neurotrophic tyrosine kinase receptor type 1; PDGFR: platelet derived growth factor receptor; Eph: ephrin receptor; VEGFR: vascular endothelial growth factor receptor; Trbl: tribbles; CDKL: cyclin-dependent kinase like; DYRK: dual specificity tyrosine regulated kinase, Wnk: with no lysine (K)' kinases.

**Figure 7:**
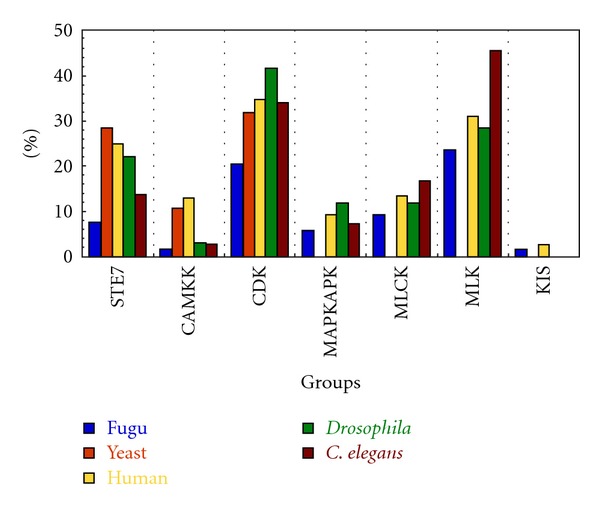
Percentage distribution of subfamilies that are under-represented in various model organisms. MLK: mixed lineage kinase; MAPKAPK: mitogen- activated protein kinase- activated protein kinase; MLCK: myosin light chain kinase; CDK: cyclin dependent kinase; CAMKK: calmodulin dependent protein kinase kinase; KIS: kinase interacting with stathmin; Ste7: sterile7.

Though the group-wise distribution is quite comparable we wanted to explore at the level of sub-families to see if the same trend is observed. Interestingly, there are certain subfamilies in which the representation by fugu kinases is noticeably higher or lower than those of other model organisms considered. The percentage-wise distribution of these kinase subfamilies has been indicated in Figures 6 and 7.  Figure 6 indicates distribution of kinases for 15 subfamilies in which fugu kinases occur in higher frequency with DYRK being highest and those of tyrosine kinase group generally being highly represented. Likewise Figure 7 shows distribution of kinases for 7 subfamilies in which fugu kinases occur at a lower frequency with CAMKK and KIS subfamilies being least. The functions of each of the proteins have been indicated in Table 2. Mutations or change in expression levels of these proteins has been implicated in various cancers (see the publications cited in Table 2). Also, profound differences in the number of paralogous proteins in two organisms could result in diverse outputs resulting in distinct features in the molecular processes in the two organisms [62]. The percentage distribution of each of the subfamilies for all the organisms considered can be obtained from Supplementary data file 2.

**Figure 8:**
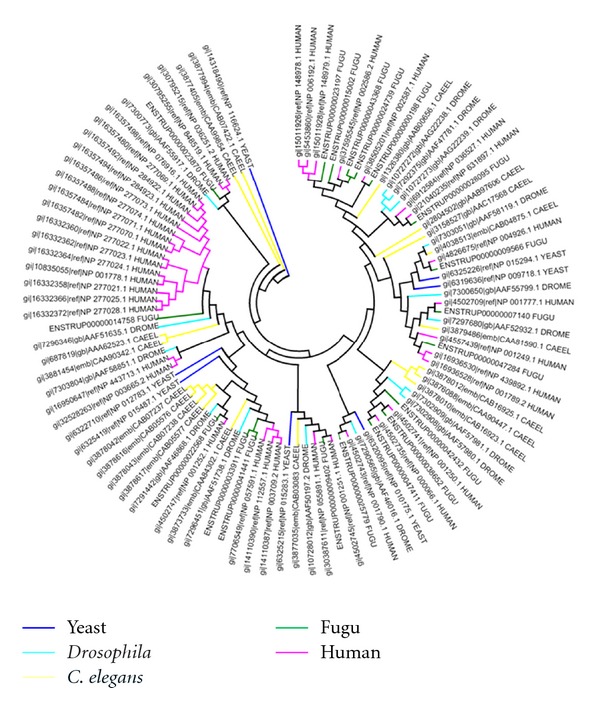
Dendrogram depicting clustering of kinases of CDK sub-family from fugu, human, *Drosophila,* and *C. elegans*.

**Figure 9:**
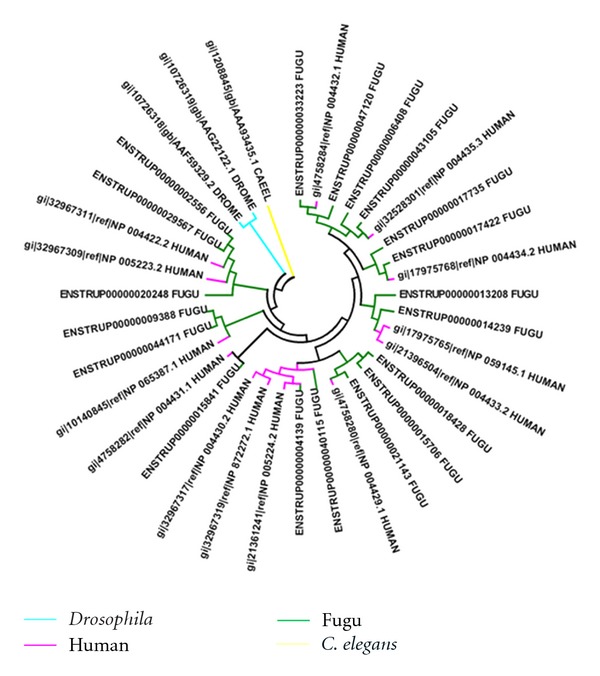
Dendrogram depicting clustering of kinases of ephrin receptor subfamily from fugu, human, *Drosophila*, and *C. elegans*.

**Figure 10:**
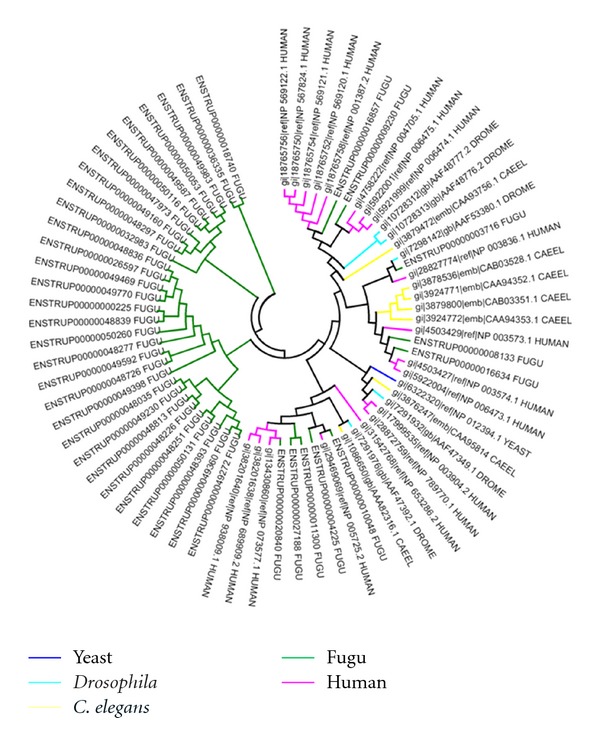
Dendrogram depicting clustering of kinases of dual specificity tyrosine regulated kinases (DYRKs) subfamily from fugu, human, yeast, *Drosophila,* and *C. elegans*.

We have analyzed the nature of clustering of these fugu kinases with respect to those in other organisms. For most sub-families, fugu kinases group quite closely to those of human and in a few cases with other organisms. A similar trend is observed in the underrepresented sub-families with different clusters being observed that are not organism specific. The example of CDK subfamily is depicted in Figure 8. In each of the clusters human kinases dominate, with few representatives from fugu. Since paralogous proteins perform diverse functions, their presence in large numbers is indicative of high functional diversity [62]. Therefore, it appears that paralogous CDKs in humans show larger functional diversity than fugu CDKs which are fewer in number. However, in the highly represented subfamilies of fugu kinases, apart from the above trend few kinases cluster separately suggesting higher divergence in a subset of DYRK kinases.  Figure 9 shows the example of ephrin receptor family. In case of the DYRK family, 30 out of 40 kinases cluster separately (Figure 10). We then explored if this is a fish-specific trait. Fortunately another fish (zebrafish) genome has been sequenced and the list of zebrafish kinases are available in the KinG database [43]. However, the zebrafish has a large kinome (~900 kinases). Analysis on these shows that in most cases the fugu kinases do not cluster closely with those of zebrafish (supplementary data file 3). We performed a BLAST [37] search against the SWISSPROT database to identify the nearest homologs for these kinases in other organisms irrespective of the genome being sequenced fully or not. It was observed that, in the sequences which cluster close to human kinases, the percentage identity of those sequences with that of human counterpart is greater than 60%. Nevertheless, the kinases belonging to the fugu-specific cluster indicate low sequence identity with that of human and relatively higher identity of about 40% with that of certain lower eukaryotes like *Xenopus tropicalis, Dictyostelium discoideum,* and fungal species (supplementary data file 4). These may be fugu-specific sequences which are functionally divergent compared to those paralogs which show significant similarity to the human sequences. Similar expansions have been seen in other organisms as well [63] that help the organism to adapt to its environment.

### 3.2. Domain Combinations

**Figure 11:**
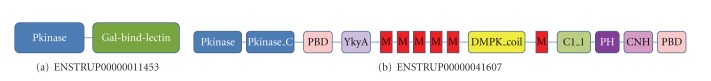
Unusual domain combinations. (a) Protein kinase tethered to a galectin binding domain. (b) Yka_A domain associated with protein kinase domain long with PBD, YkyA, M repeats, DMPK_coil, C1_1, PH, CNH. PBD: P21-Rho-binding domain; C1_1: phorbol esters/diacylglycerol binding domain; PH: pleckstrin homology.

The domain combinations for all the predicted kinases of fugu are provided in supplementary data file 1. Most of the domain combinations observed have been observed in various higher eukaryotes according to the Pfam database [56]. However there are 2 cases with an unusual domain combination as depicted in Figure 11.

In the first case, a galectin binding domain has been associated with protein kinase domain. The galectin domain is a carbohydrate binding domain and its function has been attributed to regulation of immunity and inflammatory responses, progression of cancer, and in specific developmental cascades [64]. These domains may function within or outside the cell. In this particular context, since there is no transmembrane component or domains which localize it to the membrane, the protein is likely to be cytosolic. This is further corroborated by the lack of any signal peptide motifs in this sequence which was analyzed using SignalP server [65]. The protein kinase domain may play a regulatory role wherein the activation state of the kinase might dictate the binding abilities of the galectin binding domain.

The second case involves a YkyA domain tethered to the protein kinase, CNH, PBD, PH, DMPK_Coil, C1_1, and the M protein repeats. Such a combination has been predicted also in another closely related fresh water species of Puffer fish,* Tetraodon nigroviridis,* however, without the YkyA domain. The YkyA domain has been reported only in bacterial species and is a putative lipoprotein binding domain occurring as a single-domain protein which aids in virulence [66]. It is likely that this protein is localized to the membrane due to the presence of the PH domain which is a reasonable indicator for membrane localization [67]. However, the role of YkyA in fugu is unclear.

Eight fugu kinase sequences are predicted to have two protein kinase domains each containing all the prerequisites for a functional kinase. This feature is distinct from that of Janus kinase which comprises a nonfunctional kinase domain (lacking critical aspartate) apart from a functional kinase domain. Five of these 8 cases correspond to the AGC group while the rest belong to the CAMK group. Such twin kinases have been reported in other higher eukaryotes in proteins like MAPKAP-K1 [68] wherein the C-terminal kinase domain is regulatory in nature and is involved in the activation of the N-terminal kinase domain. In all these 8 cases, both the kinase domains within a protein belong to the same group and sub-family which may be indicative of a duplication event and it may act by fine-tuning the activity levels of the protein. Interestingly, in most cases the C-terminal protein kinase domain has lower sequence identity to the respective groups than the N-terminal domain.

### 3.3. MAPK Signaling Pathway in Fugu

**Figure 12:**
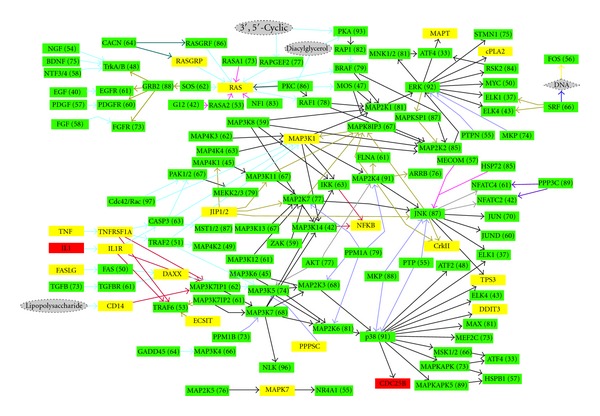
Human-fugu homologs MAPK pathway. Boxes in green represent closely related homologs of human MAPK pathway proteins present in fugu; yellow boxes indicate remote homlogs and red boxes indicate potential absence of homologs in fugu.

Given the slight unusual distribution of fugu kinases in terms of subfamilies we wanted to investigate the overall effect on a signaling pathway by considering distribution of all the proteins involved (including nonkinases) in the pathway. Although we did not observe any significant skewing in the clustering of the MAPK subfamily we chose to work on the MAPK pathway as the proteins involved in this pathway are extremely well characterized in other eukaryotes, especially for humans. Both kinases and non-kinases (including upstream factors and downstream effectors) were considered for the analyses. The extent of sequence similarity is an approximate indicator of the similarities of the functions of human and fugu proteins involved in MAPK pathway. Results are depicted pictorially in Figure 12. The cases with high sequence identity (>30%) and coverage (>70%) (compared to human proteins) have been represented as green boxes. These indicate the presence of functional counterparts, which are present in approximately 90% of the cases. Interestingly in a few cases (represented as yellow boxes) the sequence similarity levels reveal distant homologs suggesting differences in molecular events. These include proteins which act as ligands (FAS, TNF) and receptors (those of IL1, TNF, and certain lipopolysaccharides) which are mainly implicated in signaling pathways of immune system along with few proteins of the three tier MAPK (MAPKKK, MAPKK, MAPK) cascade. It also includes Ras and its activating protein RasGrp. This is especially interesting because Ras is a key component involved in GTP exchange which is a crucial step in activating the MAPK cascades. Even more glaringly, two proteins (IL1, CDC25b) do not have any identified homologs in fugu. Potential absence of IL1 is especially interesting because its absence indicates alternate ligands which are able to bind to its receptor. The other protein without any detectable homolog is CDC25b, which is a phosphatase involved in activating CDK. Absence of CDC25b may be viewed in the light of the fact that CDK subfamily is underrepresented in the fugu genome.

## 4. Conclusions

The current analysis on the fugu genome indicates a kinase repertoire of ~3% of the total genome which is slightly higher than an average of around 2% in the other model organisms. All the groups of eukaryotic protein kinases are found to be present in this genome with comparable numbers to that of humans. The observed distribution of few kinase subfamilies is fugu specific. The presence of unique domain combinations gives an insight into possibility of new regulatory functions of kinases. Finally, the similarity to signaling pathways in human may not only provide a platform for studying signaling systems but can also be used in kinase drug screening.

## Supplementary Material

Supplementary data 1 provides a list of all the 519 protein kinase like sequences encoded in the fugu genome along with their subfamily distribution, domain combination and information on presence of transmembrane helices. Supplementary data 2 presents percentage-wise distribution of all the subfamilies of protein kinases encoded in fugu genome in comparison to other model organisms. Supplementary data 3 shows a dendrogram depicting clustering of kinases of Dual specificity tyrosine regulated kinases (DYRK) subfamily from fugu, human, yeast, Drosophila, C.elegans and Zebrafish. Supplementary data 4 gives the results of BLAST search, in SWISSPROT database, for each of fugu protein kinase sequences belonging to DYRK subfamily.

## Figures and Tables

**Table 1:** Distribution of kinases of fugu, yeast, human, Drosophila, C. elegans into various Hanks and Hunter groups. Numbers indicated as percentage.

Groups	Organism				
	Fugu	Yeast	Human	*D. melanogaster*	*C. elegans*
AGC	13.48	15.23	11.72	17.35	11.04
CMGC	17.91	20.95	19.82	16.43	15.82
CAMK	16.57	22.85	16.72	15.51	12.53
STE	7.51	13.33	8.9	8.21	6.56
CK1	1.54	3.8	1.8	5.45	19.4
TK	18.68	0	20.34	15.52	20.59
TKL	7.32	0	7.2	6.39	3.28
Others/Unclassified	16.95	26.66	13.27	15.06	10.74

**Table 2:** Kinase sub-families with abnormal distribution in fugu. Overall function of each of the family is also indicated.

Family	Function
*Highly represented families*	
JakA	Receptor tyrosine kinase. Activates STAT involved in interferon signaling [7]
Lmr	Receptor tyrosine kinase involved in apoptosis [8]
PDGFR	Receptor tyrosine kinase activating factors for growth, differentiation, development [7]
Eph	Receptor tyrosine kinase component of developmental pathways [9]
InsR	Receptor that binds insulin and has a tyrosine-protein kinase activity [10]
PIM	Phosphorylating chromatin proteins and controlling transcription [11]
CDKL	Mediates phosphorylation of MECP2 [12]
Trio	Guanine nucleotide exchange factors that mediate cell invasiveness [13]
DYRK	Directs cellular response to stress conditions and also implicated in neuropathological characteristics of Down's syndrome [14, 15]
Trk	Receptor for neurotrophin-3 (NT-3) [16]
Src	Nonreceptor protein tyrosine kinase that plays pivotal roles in numerous cellular processes such as proliferation, migration, and transformation [17]
VEGFR	The VEGF-kinase ligand/receptor signaling system plays a key role in vascular development and regulation of vascular permeability [18]
Sgk496	Induces both caspase-dependent apoptosis and caspase-independent cell death [19]
Trbl	Interacts with MAPK kinases and regulates activation of MAP kinases [20]
Wnk	Controls sodium and chloride ion transport [21]

*Underrepresented families*	
KIS	Function unknown
MLCK	Calcium/calmodulin-dependent enzyme implicated in smooth muscle contraction via phosphorylation of myosin light chains [22]
MLK	Involved in the JNK pathway [23]
MAPKAPK	Integrative element of signaling in both mitogen and stress responses [24]
CDK	Involved in regulation of cell cycle by binding to cyclins [25]
CAMKK	Calcium/calmodulin-dependent protein kinase that belongs to a proposed calcium-triggered signaling cascade involved in a number of cellular processes [26]
Ste7	MAP2K homologous to yeast Ste7 [27]
