# Supplementary material for: Repertoire of Protein Kinases Encoded in the Genome of Takifugu rubripes
Source: Comp Funct Genomics. 2012 May 14;2012:258284. doi: 10.1155/2012/258284 (PMC3359783; doi:10.1155/2012/258284)
Supplement: Supplementary file 3 [file 258284.f3.pdf]

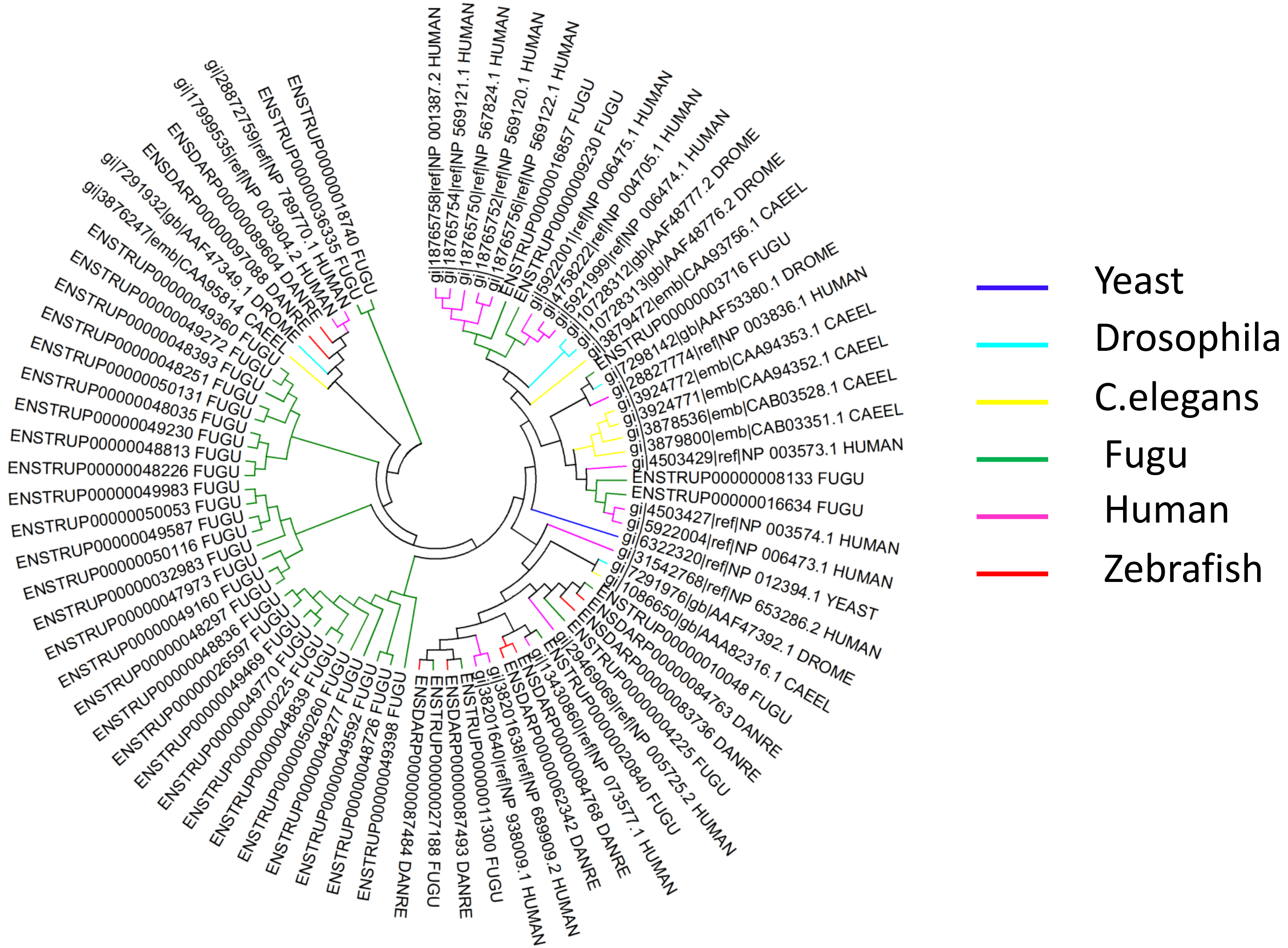

Supplementary file 3

Dendrogram depicting clustering of kinases of Dual specificity tyrosine regulated kinases(DYRK) subfamily from fugu, human, yeast, Drosophila, C.elegans and Zebrafish
